# DNA context represents transcription regulation of the gene in mouse embryonic stem cells

**DOI:** 10.1038/srep24343

**Published:** 2016-04-14

**Authors:** Misook Ha, Soondo Hong

**Affiliations:** 1 Samsung Advanced Institute of Technology, Samsung Electronics Corporation, Suwon 443-803, Korea; 2 Department of Industrial Engineering, Pusan National University, Busan, 609-735, Korea

## Abstract

Understanding gene regulatory information in DNA remains a significant challenge in biomedical research. This study presents a computational approach to infer gene regulatory programs from primary DNA sequences. Using DNA around transcription start sites as attributes, our model predicts gene regulation in the gene. We find that H3K27ac around TSS is an informative descriptor of the transcription program in mouse embryonic stem cells. We build a computational model inferring the cell-type-specific H3K27ac signatures in the DNA around TSS. A comparison of embryonic stem cell and liver cell-specific H3K27ac signatures in DNA shows that the H3K27ac signatures in DNA around TSS efficiently distinguish the cell-type specific H3K27ac peaks and the gene regulation. The arrangement of the H3K27ac signatures inferred from the DNA represents the transcription regulation of the gene in mESC. We show that the DNA around transcription start sites is associated with the gene regulatory program by specific interaction with H3K27ac.

Understanding gene regulatory information in DNA is a fundamental concern in biomedical research^1^,^2^,^3^ and the DNA signatures of non-coding regulatory elements have been actively investigated^4^,^5^,^6^. Yet the regulatory codes in DNA sequence context remain largely unknown^1^,^7^. Cell-type specific regulatory networks composed of gene regulating protein factors and features of the *cis*-regulatory elements may be used to infer gene regulation in a condition, thereby understanding the molecular mechanisms performed by cells for specific gene regulatory programs. Transcription start sites (TSS) regions including promoter and 5′ end of genes interact with transcription factors and transcription initiation complex including RNA polymerases^8^. Since regulation of transcription initiation is an important mechanism of gene regulation, we focus on DNA sequences around TSS to infer gene regulation from DNA.

Inside a nucleus, genomic DNAs are packed into 3D structures. Chromatin modifications^2^,^9^, transcription regulating protein factors^2^,^9^, and RNA Pol II complexes^10^ mediate the configurations of the chromatin 3D structures that bring regulatory elements, even in distant DNA segments, to the target genes for transcription regulation^11^,^12^,^13^,^14^. *Chromatin modifiers* reversibly change chromatin modification status^6^,^15^,^16^. Specifically, H3K27ac marks both active enhancers and active promoters^17^,^18^,^19^,^20^,^21^ and interacts with RNA Pol II transcription machineries^10^ and other transcription factors^21^. The complex interactions in the DNA imply that understanding a regulatory signature in DNA will directly give us full regulatory information about the genes. Therefore, we hypothesize that analyzing the primary DNA sequence around TSS will enable us to identify and untangle the explanatory factors hidden in the DNA sequence context. The differential regulation of genes from a common DNA sequence suggests that it encodes multiple contexts and reads them in diverse ways. The distinct composition of DNA base-pairs is recognized by different factors depending on the context. For example, co-operative, synergistic, or antagonistic interactions among protein factors and DNA elements are prevalent in gene regulation. Thus, we incorporate the DNA sequence context into our model based on the analyses of the DNA sequences interacting with protein factors. In this study, DNA sequence context refers to the combinatory effects of short DNA sequences analogous to words in sentences. We consider histone modifications as factors interacting with DNA because modified histones dissociate^22^ and incorporate^23^ dynamically.

We aim to understand the regulatory roles of DNA sequences on gene expression by interaction with the factors associated with transcription. Efforts have been made to identify DNA motifs associated with gene regulation^24^, and several approaches have been developed to model the DNA sequence specificities of chromatin modifications^25^,^26^,^27^,^28^, the relationships between gene expression and chromatin modifications^29^,^30^, and the relationships between chromatin modifications and transcription factor binding^31^. However, the separate components of gene regulation processes need to be assembled to understand the effects of each mechanism on other mechanisms and on whole gene regulatory systems. In this study, we develop a unified model integrating two essential biological processes: (1) the interaction of DNA sequences with the factors associated with transcription regulation, and (2) the effect of DNA sequence signatures interacting with the factors on gene regulation. We evaluate our model by comparing the predicted transcription and experimental measurements. From the results, we infer the gene regulatory systems and gain insights into the involvement of DNA sequences around TSS in transcription regulation.

By conducting association analyses in TSS, we find that H3K27ac around TSS is an informative descriptor of transcription regulation. Our model estimates the probabilities of interaction with the factors for individual 6mer DNA sequences, rather than discovering the DNA motifs of binding sites. Considering the combinatory effects of the DNA sequences and the co-operative binding property of H3K27ac as attributes, we predict co-ordination of H3K27ac around TSS. Next, we quantify the effects of the H3K27ac co-ordination around TSS inferred from the DNA sequences. Previous studies have used chromatin modifications measured from ChIP-seq to predict gene expression^30^, but we use the H3K27ac signatures around TSS inferred from the DNA as attributes of gene expression. Finally, we generate a model quantifying the effects of the DNA sequence on transcription regulation via H3K27ac. The result suggests that the DNA sequence context associated with the H3K27ac profile around TSS is involved in cell-type specific gene regulation.

## Results

### Factors associated with transcription regulation in mESC

We begin by investigating the informative features that will most efficiently represent trans-regulatory logic and the associated *cis*-regulatory sequence context encoded in a primary DNA sequence. Clearly, the performance of any probabilistic inference model depends on the features chosen. To analyze the mechanistic roles of the important protein factors, we measure their DNA binding signals using ChIP-seq (chromatin immunoprecipitation and sequencing of the bound DNA)^16^,^20^,^32^,^33^,^34^,^35^,^36^,^37^,^38^,^39^. Then we analyze the gene expression levels in mESC from publicly available RNA-seq data and investigate the position-specific impacts of the individual protein factors on transcription regulation and their usefulness as informative descriptors.

Using MIC (maximal information coefficient), we estimate the relationships of the mRNA levels and 52 factors at 21 positions spanning a 2 kbp region around the TSS in a 200 bp window. The 52 factors include chromatin modifications, chromatin modifiers, chromatin conformation regulators, transcription machineries, and transcription factors. Among the 52 factors and 21 binding site combinations around the TSS, MIC values show that H3K27ac in the 5′ side of the genomic region encoding genes relates most closely to the transcription regulation in mouse ESC (Supplementary Figure 1, Supplementary Table 1). In addition to the expected close relationship among Pol II and transcription factors with transcription, MIC values show that the H3K27ac in the genomic regions encoding genes explains gene expression more significantly than RNA Pol II in mESC. The finding suggests that the H3K27ac enrichment level in the 200 bp downstream of the transcription start site is the most representative feature of gene regulation in mESC.

**Figure 1:**
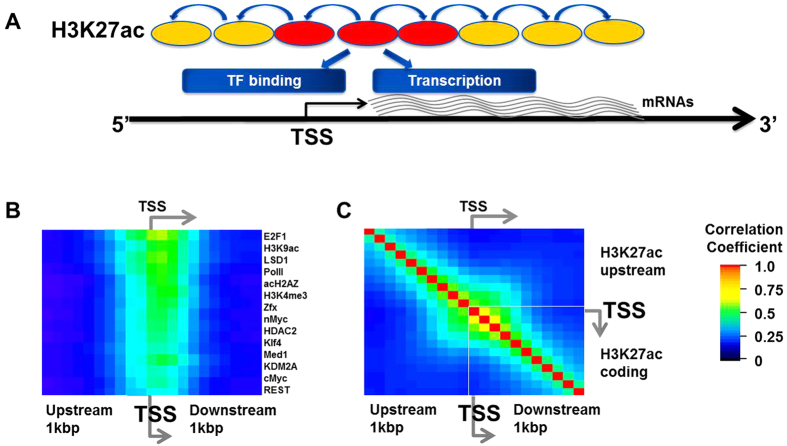
Finding representative DNA binding features of gene regulation in the coding region. (**A**) H3K27ac peak at 5′ end of coding region (red oval) is a representative feature of gene regulation in mESC. The H3K27ac in the promoter and 5′ end of coding region correlates with adjacent H3K27ac (yellow ovals) and other transcription factor binding enrichment in the region. (**B**) H3K27ac in the 5′ end of genes significantly associates with binding of the transcription factors in the genes. Color map of correlation coefficients between H3K27ac at the 5′ end of genes and other transcription regulating protein factors at each position in 200 bp sliding window with 100 bp overlap around TSS; warm color represents high association, and blue and black colors represent low association in pair-wise manner. P-values for correlations of transcription regulator binding are all less than 10^−22^. (**C**) H3K27ac enrichment around transcription start sites associate with each other. Color map of pair-wise correlation coefficients between H3K27ac at 200 bp sliding window with 100 bp overlap in 2 kbp region around TSS.

We examine the position-specific relationships among H3K27ac in the 5′ end of the genes and the factors associated with transcription regulation. To understand position-specific interaction of H3K27ac in the 5′ end of genes with other factors in distinct positions, we examine correlations of H3K27ac in the 5′ end of genes with various factors in a 200 bp window. We find that the H3K27ac in the 5′ end of the genes highly correlates with bindings of transcription regulators in the upstream and downstream of TSS (Fig. 1B). H3K27ac in the 5′ end of the gene also highly correlates with H3K327ac in the upstream of TSS (Fig. 1C), which suggests cooperative deposition of the H3K27ac nucleosomes.

Transcribed mRNA levels are most closely associated with H3K27ac around TSS among the 52 factors. H3K27ac is significantly associated with mRNA levels as well as various factors including other chromatin marks, transcription factors, and Pol II (Fig. 1A). The results imply that the H3K27ac around TSS is an informative descriptor of the gene regulation.

### H3K27ac profiles around TSS predict transcription regulation in mESC

We determine whether the H3K27ac profile around TSS can predict a gene’s mRNA level. The distribution of RNA levels shows bimodal distribution (Supplementary Figure 2B), from which we infer distributions of RNA levels of significant expression and not significantly detected. We used 1 FPKM as a threshold of significant expression levels because 1 FPKM maximizes the distance between the modes of two bell-shaped distributions. We use logistic regression. In the logistic regression model, a target variable is the probability that the gene is expressed, *P*(expression = 1) (Equation 1). As RNA levels rise, the probabilities of the gene expression also rise. We examine the correlations between the predicted probabilities of gene expression and measured RNA levels.

**Figure 2:**
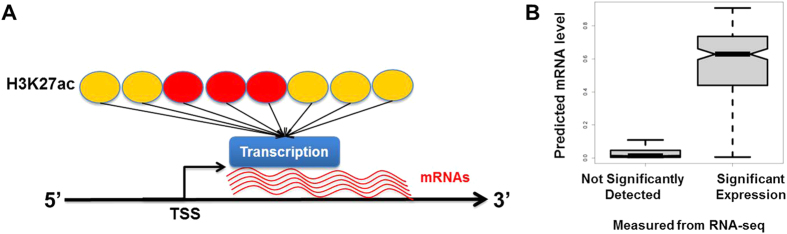
H3K27ac profile around TSS predicts gene regulation in mESC. (**A**) A model predicting the mRNA level from H3K27ac ChIP-seq data. H3K27ac ChIP-seq reading signals in 1 kbp around transcription start sites are used to develop the model inferring the mRNA levels from H3K27ac profiles in the TSS regions: 1 and 0 as significant and not significant transcription detected from RNA seq experiments. H3K27ac in the 5′ end of coding regions (red ovals) are more weighted than other H3K27ac (yellow ovals) in the regression model. (**B**) The predicted mRNA level from the H3K27ac profile around TSS highly correlates with experimental measurements using RNA-seq analysis in mESC. The vertical axis is the predicted mRNA level of the model. The left boxplot shows the distribution of the predicted mRNA levels in genes without significant transcription detected and the right boxplot shows the distribution of predicted mRNA level in genes with significant transcription detected from RNA-seq experiment. The predicted mRNA levels in genes showing significant expression in RNA-seq experiment are significantly higher than those of not significantly detected mRNAs. Wilcoxon rank sum test between significantly expressed and not significantly detected, W = 255195.5, p-value < 10^−22^.

We generate a model inferring a mRNA level from the H3K27ac profile using 50% of the genes (Fig. 2A) and validate the model by applying it to the remaining 50% of the gene sets that are not used for modeling. The results show that the predicted mRNA levels are significantly consistent with the mRNA levels measured from the RNA-seq in mESC (PCC between predicted and measured mRNA levels = 0.72, p-value ≈ 0; Fig. 2B). The H3K27ac arrangements in the gene efficiently distinguish genes showing significant expression from genes not significantly detected in mESC (Wilcoxon rank sum test between genes showing significant expression and genes not significantly detected, W = 255195, p-value ≈ 0; Fig. 2B, Supplementary Figure 3). The results further support that the H3K27ac profile in the gene is an informative feature of gene regulation in mESC.

### Inference of H3K27ac signatures in the DNA sequence and validation

To understand the DNA sequences associated with H3K27ac, we investigate compositions of DNA base-pairs in H3K27ac peak sites and H3K27ac depleted sites around TSS. We generate relative association model of H3K27ac specificity for individual 6 bp DNA sequences around TSS. All possible 6 bp DNA sequences are 4^6^ (=4096) combinations of “A”, “T”, “G” and “C”. Due to the limited number of sequences used for inference, the interaction intensities of 7mer or longer DNA sequences are not sufficiently observed in the limited numbers of the DNA sequences around TSSs. As such, the uncertainty of inference increases with the DNA sequences longer than 6 bp. In addition, using the longer DNA sequences in the model increases the number of variables and the model complexity exponentially. Therefore, we use 6mer to examine the association with H3K27ac for individual short sequences.

**Figure 3:**
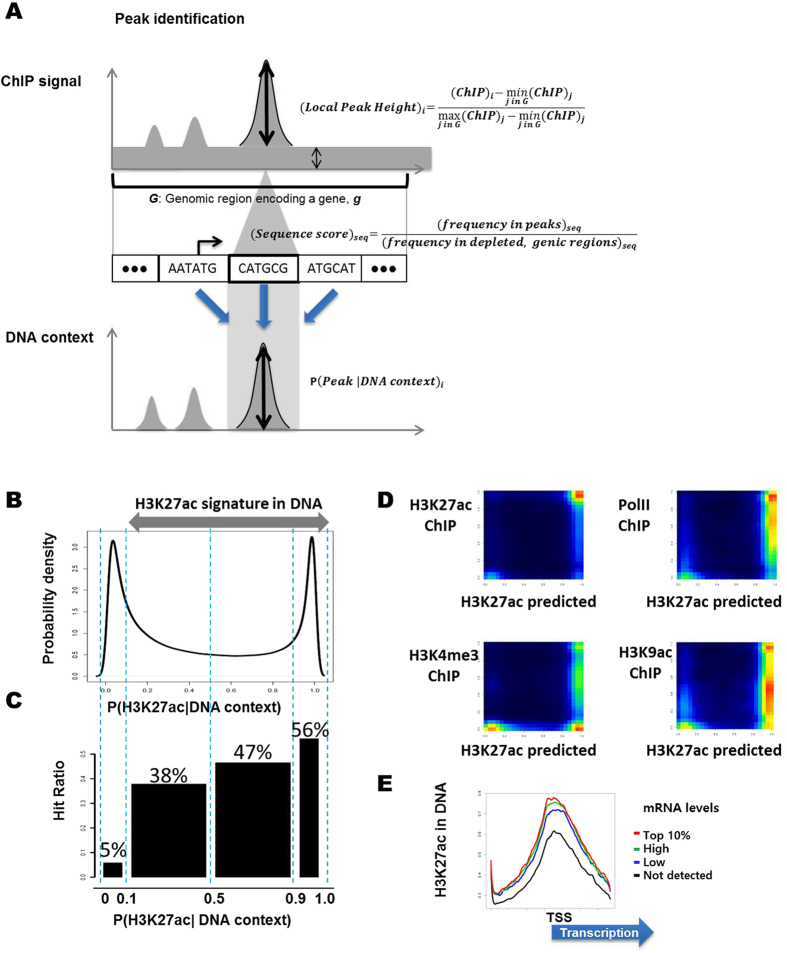
Inferring H3K27ac signatures from the DNA sequence. (**A**) H3K27ac peaks around TSS are identified from local H3K27ac ChIP-seq read enrichment compared to the neighboring genomic region. The 6mer sequence composition in the H3K27ac peaks normalized with 6mer frequency in H3K27ac depleted regions around TSS is used for estimating the probability of forming a H3K27ac peak at a locus. (**B**) The distribution of the inferred H3K27ac peak height from the DNA sequence. (**C**) The hit ratios of H3K27ac ChIP-seq peaks that correlate with the inferred H3K27ac peak heights from the DNA suggest that the H3K27ac signature in the the DNA distinguishes H3K27ac peaks. (**D**) The H3K27ac signature in the DNA is specific to H3K27ac in mESC and correlates with RNA polymerase II and other mESC-specific transcription regulators. The predicted H3K27ac peak heights from the DNA sequence context significantly correlates with H3K27ac ChIP-seq read enrichment. Color represents number of loci in the pixel of the predicted H3K27ac enrichment and the local ChIP-seq peaks of H3K27ac, H3K9ac, Pol II, H3K4me3, E2F1, and KLF4 respectively. PCC H3K27ac sequence signature vs. H3K9ac ChIP-seq = 0.242; H3K27ac sequence signature vs. Pol II ChIP-seq = 0.278; H3K27ac sequence signature vs. H3K4me3 = 0.295; H3K27ac sequence signature vs. KLF4 ChIP-seq = 0.186; H3K27ac sequence signature vs. E2F1 ChIP-seq = 0.185. All p-values < 10^−22^. (**E**) Distribution of H3K27ac sequence signature around transcription start sites. The significantly expressed genes associate with significantly higher H3K27ac signatures in their DNA than the genes associated with not significantly detected RNAs do. Top 10%, High, Low, and Not detected mRNA levels are top 10%, greater than average, lower than average, and not significantly detected levels in the RNA-seq experiments.

Here, we define H3K27ac enriched regions as the 2 kbp region around TSS with at least 5 ChIP-seq reads mapped. In the H3K27ac enriched regions, we count each 6mer at a locus as peak height which represents enrichment compared to adjacent sequences and the probability that the 6mer locus is in H3K27ac peak (Fig. 3A). For example, a 6mer sequence at peak position is counted as 1 and a 6mer sequence around a peak is counted as a probability between zero and 1 (Equation 2).

To estimate the relative specificities of H3K27ac for individual short DNA sequences, we compare the 6mer sequence frequencies in H3K27ac peak sites with the frequencies in H3K27ac depleted sites around TSS, respectively (Equation 4 and Equation 5). Since the gene-rich regions show high GC content and different compositions from other genomic regions, normalization with the DNA composition in depleted regions around TSS adjusts the GC content and estimates the tendency of H3K27ac peak formation of a sequence over the H3K27ac depleted sites around TSS. The 6mer sequences enriched in H3K27ac peaks around TSS in mESC include “TAAAGC”, “ATGCGC”, “CTTGTC”, “CGGTGT”, and “GCGCGT” which are part of the known transcription factor motifs in Zfx, Oct4, Nanog, KLF4, and Myc, respectively (Supplementary Table 2 and Supplementary Figure 4)^40^. The sequence “AAAAAA” associated with nucleosome-free regions is enriched in both H3K27ac peaks and depleted sites around TSS.

Using the sequence specificity of mESC H3K27ac peaks, we build a probabilistic model inferring the H3K27ac signatures from the DNA sequence context around TSS. We define the DNA sequence context as the combinatory information derived from the properties of the target sites and other sites. Our computational model calculates the probabilistic enrichment of H3K27ac at a site by combining the probabilities of adjacent sites, their effects, and the base pair composition at the site. In this way, the effect of the H3K27ac enrichment at a position propagates to other sites including adjacent loci^41^ (Equations 5, 6, 7). Thus, the model includes the cooperative properties of the H3K27ac peaks and considers the association among proximal sites. We find high performance of this probabilistic model compared to H3K27ac ChIP-seq peaks (Supplementary Figure 5A,B).

We determine how efficiently our DNA sequence-based model can distinguish the H3K27ac ChIP-seq peaks from the dips in the genomic regions around TSS. We classify loci around TSS by the probabilities of H3K27ac inferred from the DNA sequence. The very high, high, medium, and low DNA sequence signatures of H3K27ac refer to probabilities of H3K27ac greater than 0.9, between 0.9 and 0.5, between 0.5 and 0.1, and less than 0.1, respectively (Fig. 3B). Among the H3K27ac peak sites of H3K27ac ChIP-seq, 56%, 47%, 38% match very high, high, and medium signatures of H3K27ac inferred from DNA, respectively, whereas 5% of H3K27ac ChIP-seq peaks match low sequence signature of H3K27ac (Fig. 3C). As a rough hit ratio, the model correctly detects 95% of the H3K27ac ChIP-seq peak sites as H3K27ac enriched sites in the very high, high, and medium signature regions, respectively, and 5% of the H3K27ac ChIP-seq peak sites as H3K27ac enriched sites in the low signature region. The results suggest a high efficiency of the sequence-based inference.

Next, we compare the probability of H3K27ac enrichment and the height of the ChIP-seq peaks in ESC. We find that the probabilistic H3K27ac enrichment inferred from the DNA sequence context significantly correlates with the H3K27ac ChIP-seq peaks. Specifically, a high probabilistic H3K27ac sequence context is associated with the H3K27ac ChIP-seq peaks (PCC = 0.32, p-value < 10^−22^; Fig. 3D). The correlation analysis between predicted and measured values is one of the strictest measures of a predictive model. We conduct Receiver Operating Characteristic (ROC) Curve Analysis to further assess the performance of H3K27ac prediction from the DNA sequences around TSS (Supplementary Figures 5A,B). In an ROC curve, the true positive rate (sensitivity) is plotted with the false positive rate (1- specificity) for different positivity thresholds of a H3K27ac enrichment. The AUC (Area Under Curve) is a measure of how well a predictive model can distinguish between loci with and without H3K27ac enrichment signals. The true positive rate is the proportion of the loci with enriched H3K27ac ChIP-seq reads mapped among the predicted H3K27ac sites with different thresholds, and the false positive rates are the proportion of H3K27ac ChIP-seq reads not detected among the predicted H3K27ac sites. The AUC value is 0.89 with p-value < 10^−16^ suggests that the model based on DNA sequences efficiently distinguishes H3K27ac around TSS. In summary, we conclude that our model is significantly associated with H3K27ac local peaks around TSS.

### H3K27ac peaks inferred from the DNA context are specific to the H3K27ac ChIP-seq peaks

Further examining the relationships of the H3K27ac signatures in the DNA contexts with other transcription regulators, we find that the H3K27ac signatures inferred from DNA contexts correlate more closely with the H3K27ac ChIP-seq signals than the ChIP-seq signals of Pol II, H3K9ac, H3K4me3, E2F1, Klf4, and other ESC-specific transcription factors (PCC H3K27ac sequence signature vs. H3K9ac ChIP-seq = 0.242; H3K27ac sequence signature vs. Pol II ChIP-seq = 0.278; H3K27ac sequence signature vs. H3K4me3 = 0.295; H3K27ac sequence signature vs. KLF4 ChIP-seq = 0.186; H3K27ac sequence signature vs. E2F1 ChIP-seq = 0.185; Fig. 3D). The H3K27ac signature in the DNA context efficiently recapitulates the H3K27ac ChIP-seq signals. Like the H3K27ac ChIP-seq peaks in mESC, the H3K27ac signatures in the DNA preferentially associate with nucleosome depletion and the ESC-specific transcription activator enrichment (Supplementary Figure 6). We note that the significantly expressed genes in mESC associate with H3K27ac signatures inferred from the DNA in the 5′ end of the genes (Fig. 3E).

The 5′ end of genes shows a higher probability of H3K27ac than the upstream regions and other gene-coding regions (Fig. 3E). The 6 bp sequences enriched in H3K27ac peaks around TSS include sequences of specific combinations with the start codon, “ATG”, which form known binding motifs of DNA-binding proteins. Specifically, the 6mer sequences, “ATGNNN” enriched in H3K27ac are found in KLF4 binding motifs such as “AGGATGGGGG” and “ATGGAGTGGC”, Myc binding motifs such as “CCATATGGGG” and Zfx binding motifs such as “CTAGCCCATGCCTG”.

In summary, we conclude that the sequence-based model significantly associated with the H3K27ac peaks around TSS. The results also suggest that mESC-specific H3K27ac peaks associate with distinct DNA sequence features.

### The mESC H3K27ac signature in the DNA context is associated with the ESC-specific gene regulatory program

**Figure 4:**
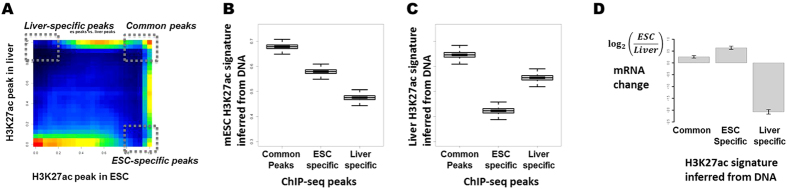
Sequence signature encoding mESC-specific H3K27ac confers mESC-specific gene expression. (**A**) Differentiation of the H3K27ac around TSS in adult liver cells from mESC. The vertical and horizontal axes represent H3K27ac peak heights in adult liver cells and mESC, respectively. Common and cell-type specific H3K27ac peaks among mESC and adult liver cells are identified as illustrated. (**B,C**) mESC-specific H3K27ac peak sites encode high levels of mESC H3K27ac signatures and low levels of liver H3K27ac signature in the DNA sequences compared to liver-specific H3K27ac peaks. Liver-specific H3K27ac peak sites associate with high levels of liver-H3K27ac signatures and low levels of mESC-H3K27ac signatures in the DNA. The vertical axes are levels of H3K27ac signatures encoded in DNA of mESC (**B**) and liver (**C**), respectively. The boxplots show the distribution of H3K27ac signatures in DNA at common, mESC-specific, and liver-specific H3K27ac peak sites, respectively. (**D**) Log2 fold changes of RNA levels in mESC from mouse liver cells are measured in genes associated with high H3K27ac signatures in their DNA sequences around TSS. Common refers to genes associated with the probability of H3K27ac greater than 0.5 in both mESC and liver cells. mESC specific and liver specific refer to the genes associated with the probability of H3K27ac greater than 0.5 only in the mESC and liver cells, respectively, but less than 0.1 in the other cell-type.

To test whether the mESC-H3K27ac signature represents mESC-specific H3K27ac profile in the DNA, we analyze the H3K27ac ChIP-seq data of mouse adult liver cells that are differentiated from mESC. Comparing the H3K27ac peak heights from H3K27ac ChIP-seq in the two cell-types illustrates dynamic local changes of H3K27ac levels in the genomic regions encoding genes between mESC and the adult liver cells. Based on the differentiation of local peak heights of H3K27ac peaks in mESC and the liver cells, we group the loci into mESC and liver cell-specific peaks, and common peaks among mESC and the liver cells (Fig. 4A). Like the H3K27ac peaks common to mESC and the adult liver cells, the mESC-specific peaks show significantly high levels of the mESC-H3K27ac signatures in DNA compared to the liver-specific H3K27ac peaks (p-value ≈ 0, Wilcoxon rank sum test; Fig. 4B). We note that the mESC-specific peaks show significantly low levels of the liver-H3K27ac signatures in DNA (p-value ≈ 0; Fig. 4C). The results suggest that the H3K27ac signatures in DNA represent the H3K27ac peaks specifically in mESC.

To explore the cell-type specificity of the H3K27ac signature in DNA, we infer a H3K27ac signature of the adult liver cells in DNA using the H3K27ac ChIP-seq data in adult liver cells. We find that the liver-specific H3K27ac peaks show a significantly high level of the liver-H3K27ac signatures compared to ESC-specific H3K27ac ChIP-seq peaks (p-value ≈ 0; Fig. 4C).

We segment the genomic regions around TSS into common, mESC, and liver-specific H3K27ac signatures. The sites with common H3K27ac signatures in mESC and mliver are defined as the loci with probability of H3K27ac greater than 0.5 in both mESC and mliver. The sites of mESC-specific signatures are defined as the loci with probability of H3K27ac greater than 0.5 only in mESC but less than 0.1 in liver. Likewise, the sites of mouse liver-specific signatures are defined as the loci with probability of H3K27ac greater than 0.5 only in liver but less than 0.1 in mESC. We observe that the DNA encoding common, mESC, and liver-specific H3K27ac signatures are associated with the common, mESC, and liver-specific H3K27ac peaks measured using ChIP-seq experiments (Supplementary Figures 7A,B). The results suggest that the H3K27ac signatures in DNA associate with the dynamic regulation of H3K27ac in a cell-type-specific manner.

Finally, to determine any relationship between the cell-type specific H3K27ac signature in DNA and gene regulation, we analyze the gene expression changes in mESC and the adult liver cells. The genes encoding common H3K27ac signatures in DNA show significant expression levels in both mESC and the adult liver cells, whereas the genes encoding the mESC-specific signatures of H3K27ac in DNA show mESC-specific up-regulation (Fig. 4D). Likewise, the genes encoding the liver-specific H3K27ac signatures show liver-specific expression. These findings suggest that cell-type specific H3K27ac signatures in DNA confer cell-type specific activation of gene expression. In summary, we conclude that the H3K27ac signatures of H3K27ac in mESC represent the gene regulatory codes of mESC.

### Predicting gene regulation program from the H3K27ac sequence signature

We examine whether the arrangement of the H3K27ac signatures in the DNA is a representative feature of transcription status in mESC. We generate a probabilistic model inferring mRNA levels from the H3K27ac signatures in DNA around TSS. The computational model uses the H3K27ac signature in the DNA at each position of 2 kbp around the transcription start sites as input attributes. Applying the artificial neural network to the 334 position-specific H3K27ac signatures in the DNA context allows us to efficiently predict the transcription regulation in mESC.

**Figure 5:**
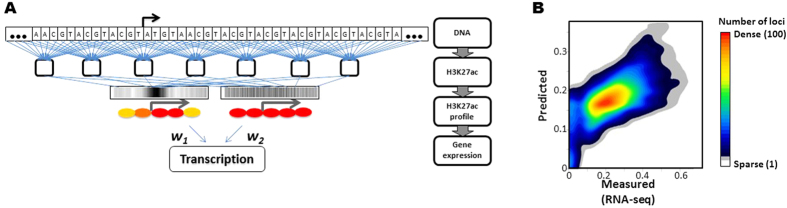
Predicting transcription regulation from the H3K27ac sequence signature. (**A**) The two steps needed to infer gene regulation from the DNA. In our model, the DNA composition is represented by the probabilistic levels of the H3K27ac signatures in mESC. Based on the sequence features of the H3K27ac, the H3K27ac signatures encoded in the DNA are predicted. The mRNA levels in mESC are predicted by combining the effects of arrangements of the H3K27ac signatures encoded in the DNA. The probabilistic H3K27ac signatures in 6 bp resolution are used for a predictive model of gene regulation in mESC. (**B**) Performance of the predictive model. The predicted mRNA levels (vertical axis) highly correlate with the mRNA levels measured using RNA-seq (horizontal axis) in mESC. Spearman’s rank correlation coefficient = 0.9, p-value ≈ 0. Color in density plot represents number of loci.

We specifically use a neural network to model the effects of position-specific coordination of the predicted H3K27ac levels on gene regulation. In a neural network, the combinations of input variables comprise hidden variables, and thus, the effects of hidden variables on target variables can be estimated^42^. We use Autoencoder^43^ to reduce the 334 dimensions and understand the intrinsic combinations of position-specific probability of H3K27ac. The two coordinates explain 99.7% of the variance of H3K27ac signatures. Therefore, with 0.3% loss of information, we construct two variables which are combinations of position-specific H3K27ac signatures. The neural network model compresses the 334 position-specific levels of the H3K27ac signature in the DNA context to two variables that are the combinatory effects of the signals and then estimates the combinatory effects of the distinct arrangements of the H3K27ac signatures on transcription regulation. The coordinate explaining the most variations of the H3K27ac signature in the DNA associates with the high peaks of H3K27ac signatures in the 5′ side of the genes, whereas the other coordinate is the relatively even combinations of the H3K27ac sequence signatures from the promoter to the 5′ side of the gene. The coordinates of the H3K27ac sequence signatures identified from the model are consistent with the H3K27ac ChIP-seq signals associated with gene regulation in mESC (Fig. 5A).

We generate a predictive model using 10,000 randomly selected sets of genes and validate its general performance using the remaining sets of genes that are not used for model building. We find that the predicted mRNA levels and the measured mRNA levels using RNA-seq experiments significantly correlate (Spearman’s rank correlation rho = 0.9, p-value ≈ 0; Fig. 5B). We also compute the Area Under curve (AUC) of the ROC (receiver operating characteristic) to further assess the model’s performance. The average AUC value is 0.89, which suggests that the predictive model of gene expression from the DNA sequences efficiently predicts mRNA levels (Supplementary Figure 8A,B). The results show that the H3K27ac signatures inferred from DNA efficiently represent the gene regulations in mESC.

Notably, we observe false positive prediction (i.e., the genes that are not significantly detected by RNA-seq but predicted to be significantly expressed due to the signatures in the DNA around TSS). We examine whether false prediction of H3K27ac from DNA is associated with false inference of gene expression from H3K27ac signatures in DNA. Since we predict gene expression levels from H3K27ac signatures in DNA, the absence of H3K27ac ChIP-seq signals in the genes with high probability of expression stands for false positive prediction of H3K27ac from DNA. Likewise, H3K27ac ChIP-seq signals in the genes with low probability of expression stands for false negative signatures of H3K27ac in DNA. Among genes with significant expression detected and high H3K27ac signature in DNA, 33% are associated with false positive signatures in DNA, whereas 67% of genes with no expression detected and high H3K27ac signature in DNA are associated with false positive H3K27ac signatures in DNA around TSS (Supplementary Figure 9). The genes with false positive H3K27ac signatures are significantly enriched in the genes with false positive inference of gene expression (Chi-squared test, χ^2^ = 109.6, degree of freedom = 1, p-value ≈ 0). This result suggests that the performance of the model finding H3K27ac signatures in DNA affects the performance of the model inferring gene expression. The result also suggests that our model is limited to explain gene regulatory mechanisms via H3K27ac.

In summary, we conclude that the arrangements of the H3K27ac signature in the DNA context predict gene regulation in mESC. We suggest that the DNA contexts associated with H3K27ac arrangements around TSS can be used to interpret context-dependent *cis*-regulatory codes in the gene for the gene regulation in mESC.

## Discussion

This study describes a workflow to infer the gene regulation in a cell-type encoded in DNA around TSS. H3K27ac is known to mark active promoter and active enhancer^17^,^18^,^19^,^20^,^21^. The association analyses of position-specific H3K27ac around TSS and the gene expression show that H3K27ac in the 5′ side of the gene is positively correlated with binding of transcription factors and Pol II at the promoter. The H3K27ac around TSS including promoter and 5′ side of genes significantly explains variations of gene expression in mESC. The results suggest that H3K27ac around TSS including 5′ end of genes mark actively transcribed genes. The DNA sequences around TSS show differential affinities with H3K27ac in mESC and liver cells. The H3K27ac-specific sequences around TSS in mESC include known binding motifs of ESC-specific transcription factors. Thus, we infer the H3K27ac arrangement around TSS based on the DNA sequences around TSS. The DNA sequence signatures of H3K27ac around TSS are significantly correlated with mRNA levels of the genes in mESC. We conclude that the DNA sequences around TSS are involved in transcription regulation in mESC by differential interaction with H3K27ac and transcription factors.

Notwithstanding the limitation to the DNA around TSS, the workflow described in this study shows that the DNA sequence composition interacting with the histone modifications is an effective attribute for the inference of the gene regulation in a cell-type. Technological advances enabling accurate, rapid identification will allow gene regulatory information from DNA around TSS to be more widely applied, because DNA sequences encoding genes are more extensively sequenced than other repeat-rich non-coding regions^44^.

Future work will focus on connecting genes to their distant-acting regulatory elements across the genome. Although distal regulatory elements are important for phenotypes, target genes of distal regulatory elements are rarely known. Specifically, distal enhancers act independently of orientation and distance of the target genes. Incorporating the interactions among genes and non-coding regulatory elements should improve predictive performance of the gene regulation from the whole genome.

## Methods

### ChIP-seq data sources and mapping to mouse genome

Chromatin modification ChIP-seq for H3K4me1, H3K27ac, H3, H3K4me3, p300 for mESC and mouse adult liver cells was obtained from Creyghton *et al.*^20^ (GSE 24165)^20^. DNA methylations ChIP-seq for mC, 5hmC, 5caC, 5fC in mESC were obtained from Shen *et al.*^32^ (GSE42250)^32^. H3.3 ChIP-seq in mESC was obtained from our previous study^16^. H2AZ and acetylated H2HAZ ChIP-seq in mESC were obtained from Hu *et al.*^32^ (GSE34483)^33^. Transcription factor ChIP-Seq for Nanog, Oct4, Sox2, Smad1, E2F1, Tcfcp2I1, CTCF, Zfx, STAT3, KLF4, Esrrb, n-Myc, p300 in mESC was obtained from Chen *et al.*^34^ (GSE11431)^34^. H3, H4K20me3 H3K9me3, H3K36me3 ChIP-seq in mES was obtained from Mikkelsen *et al.*^35^ (GSE12241)^35^. KDM2A ChIP-seq in mESC was obtained from Neil P. Blackledge *et al.*^36^ (GSE21202)^36^. SUZ12, EZH2, RING1B ChIP-seq in mESC were obtained from Ku *et al.*^37^ (GSE13084)^37^. Med12, Smc1/2/3 Med1, Nipbl, CTCF ChIP-seq in mESC were obtained from Kagey *et al.*^38^. HDAC1, HDAC2, LSD1, REST (transcription repressor of neuronal genes in non-neuronal cells), COREST, Mi2b ChIP-seq were obtained from Whyte *et al.*^39^ (GSE27844)^39^.

The raw ChIP-seq data in SRA format were transformed into fastq files. The 30-50 bp sequences from the ChIP-seq data were mapped to the mouse reference genome (mm9) by perfect and unique matching without allowing any mismatch or gap using Bowtie^45^. During ChIP-seq experiments, DNA fragments of size around 150 bp were selected for sequencing. Therefore, the reads were then extended to 150 bp from their 5′ end. The H3K27ac enriched sites were validated by the multiple ChIP-seq reads mapped. H3K27ac enriched regions were defined as genomic regions having at least 5 ChIP-seq read mapped per base-pair. Among 1.87 × 10^9^ bp mappable regions in mm9 mouse genomes, 6.2 × 10^7^ bp (3.3%) were mapped with at least 5 ChIP-seq reads.

The classical p-value for the test static T(y) is mathematically formulated as:

Classical p-value = P(T(y) ≥ T(y) | 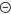), measured by the tail-area probability of a normal distribution, and parameters 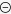.

To exclude background signals from the peak identifications, peak sites are identified only in the enriched genomic regions associated with at least 5 extended ChIP-seq reads mapped. From the experimental measurements, the proportion with at least 5 extended reads mapped are 0.033 = P(T(y) ≥ 5 | experimental data). Therefore, the genomic regions mapped with at least 5 extended reads are not likely associated with background signals. To calculate H3K27ac peak height, the 2 kbp regions around TSS containing loci with at least 5 ChIP-seq reads mapped at a base-pair were considered.

### Analysis of RNA-seq data

The raw RNA-seq of mESC was obtained from a previous study^16^. The RNA-seq analysis was performed using the Tuxedo software package with default settings. RNA-seq reads were mapped to the mouse genome (NCBI37/mm9) using Bowtie2. Tophat with default settings was used to detect splice sites. The Cufflinks software package was used to assemble transcripts based on the Refseq mRNA sequence database (mm9). A total of 48,228 transcripts were detected from two RNA-seq replicate experiments and their mean values were used for further analysis. Log2 values of the FPKM were used as target mRNA levels of the prediction models. Not significantly detected transcripts were defined as having expression levels between 0 and 1 FPKM.

### Maximal information coefficients between RNA levels and enrichment of protein factors in the genes

As genes are regulated by complex and diverse modes, both the maximal information coefficient (MIC) and Pearson’s correlation coefficients (PCC) were used to estimate the relationships of the mRNA levels and each factor at specific positions around TSS in a 200 bp window. Enrichment of a factor in a 200 bp region was estimated by the number of the ChIP-seq reads mapped to the 200 bp region. MIC measures both linear and non-linear dependency between two values (i.e., here between a protein factor and transcription regulation) and PCC shows whether or not two variables are linearly related.

MIC calculation was performed using the MINE program by Resef *et al.*^46^. The input mRNA level values were log normalized from the RNA-seq qantification.

### Differentiation of transcription between mESC and mouse adult liver cells

To examine gene expression changes between mESC and the adult liver cells, microarray data were used to measure the mRNA levels in mESC and mouse adult liver cells obtained from Creyghton *et al.*^20^ (GSE24165)^20^. The log2 values of normalized signal intensities from the previous study were used for analyses.

### A probabilistic model inferring RNA levels from H3K27ac ChIP-seq signals around the TSSs

In the logistic regression model, the probability of expression of a gene was estimated by log value of RNA-seq result of the gene divided by maximal log value of RNA levels measured in the RNA-seq experiments. The explanatory variables were local heights of H3K27ac ChIP-seq reads mapped to the 2 kbp regions around the TSS.


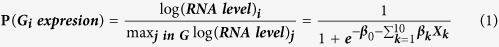


where *β*_*0*_ and *β*_*k*_ are regression coefficients and *X*_*k*_ are H3K27ac peak heights in individual 200 bp windows of the 2 kbp regions around TSS (Fig. 2).

### Calculation of H3K27ac peak heights

Local peak sites were detected as ChIP-seq read enrichment compared to adjacent sites. H3K27ac enriched regions were defined as genomic region having at least 5 ChIP-seq read mapped per base-pair.

Local peak height of H3K27ac was transformed to be between 0 and 1 by normalizing with local maximum read numbers mapped in the adjacent 2 kbp genomic region.

The local peak height at locus ***i*** was calculated as:


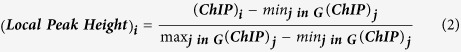


where, ***G*** is the adjacent 2 kbp region around locus ***i***

### A probabilistic model inferring H3K27ac peaks from DNA

The model aimed to distinguish between H3K27ac enriched sites and depleted sites. Therefore, a probability of H3K27ac enrichment from DNA sequence at a locus was estimated based on a method similar to a previous study predicting H3K4me3 occupation levels^41^, the difference being that in this study, the 6mer sequence enrichment in the H3K27ac ChIP-seq peaks over the H3K27ac depleted sites around TSS was estimated. Rather than restricting 147 bp as a nucleosome, this study’s model calculated the probability of forming peaks considering the sequence composition in the genomic regions around TSS and the cooperative peak formation property of H3K27ac.

The probability of a 6mer sequence S_i_ in H3K27ac peaks around TSS was estimated as a proportion of H3K27ac peak associated S_i_ loci among the H3K27ac peaks around TSS as:


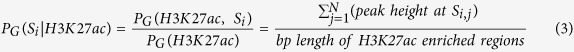


where *N* was the number of a sequence *S*_*i*_ in H3K27ac enriched regions.

Peak height at a 6 bp locus was used as the probability of peak formation at the 6 bp locus.

The probability of a 6mer sequence *S*_*i*_ in H3K27ac depletion was estimated by the proportion of sequence *S*_*i*_ in H3K27ac depletion sites of TSS regions as:


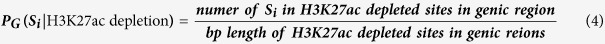


The probability that a 6 bp locus was in H3K27ac peaks was calculated by using the modified hidden Markov model to consider the adjacent H3K27ac peaks and DNA sequence contexts.

The transition probability between H3K27ac peak and dip was estimated from the proportion of H3K27ac peaks physically close to other H3K27ac peaks among the H3K27ac peaks.

To calculate the probability that a 6 bp DNA segment was in the H3K27ac peaks in a cell-type based on DNA sequence context, the effects of upstream sequences and downstream sequences of the target locus were combined by using the forward and backward procedure of HMM.

The probability that the *i*th 6 bp DNA segment was in H3K27ac peak (*M* state) based on the DNA context was normalized by the sum of all configurations (i.e., the *M* and *d* states in the model) as:


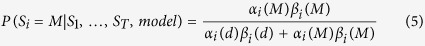


where T is the number of 6mer DNA segments in a DNA. 2004 bp around TSS and T = 334 was considered. *α*_*i*_(*M*) is the probability that the *i*th 6 bp DNA segment is in H3K27ac peak (*M* state) and the first to the *i*th 6 bp DNA sequence is observed as:


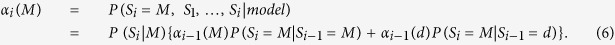


*β*_*i*_(*M*) is the probability of the sequence from *S*_*t* + *1*_ to the end of the DNA sequence when *S*_*i*_ = *M:*


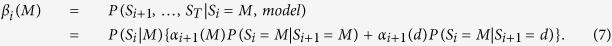


### A model predicting the mRNA level from the H3K27ac signature in a DNA sequence around TSS

The target variable of a predictive model was the mRNA levels in mESC measured using RNA-seq. The FPKM mRNA levels were transformed to LOG (FPKM + 1). The explanatory variables were the position-specific levels of H3K27ac signatures in 6 bp resolution in 2004 bp around TSS. Autoencoder, which finds a non-linear function that can reconstruct the input data by recombining input data^43^ was used to create a small number of variables that compressed the input high dimensional data by estimating the weights of the input variables and efficiently reconstructing the input data. To capture the coordinates that most efficiently summarized the 334 H3K27ac signatures, the Autoencoder was applied to the H3K27ac signatures at 334 positions in 26,000 genes. The Autoencoder results were used as the initial parameter of the artificial neural network. The Autoencoder library was implemented in R.

The whole gene sets were divided into two randomly selected sets, training sets (10,000 genes) and validation sets (3,000 genes). The weights measuring effects of the position-specific H3K27ac signatures on the mRNA levels were optimized using the training sets. The performance of the predictive model was validated using the validation sets. The correlations between the predicted mRNA levels and the mRNA levels measured using RNA-seq were calculated for validating the model’s performance. According to the central limit theorem, the Pearson correlation coefficient is a reasonable measure of the linear relationship between predicted and measured values of sufficiently large numbers of random selection of data^14^.

## Additional Information

**How to cite this article**: Ha, M. and Hong, S. DNA context represents transcription regulation of the gene in mouse embryonic stem cells. *Sci. Rep.* **6**, 24343; doi: 10.1038/srep24343 (2016).

## Supplementary Material

Supplementary Information

Supplementary Table 2
