# Supplementary material for: DNA context represents transcription regulation of the gene in mouse embryonic stem cells
Source: Sci Rep. 2016 Apr 14;6:24343. doi: 10.1038/srep24343 (PMC4831003; doi:10.1038/srep24343)
Supplement: Supplementary Information [file srep24343-s1.pdf]

Supplementary Information for

DNA context represents transcription regulation of the gene in mouse embryonic stem cells

Misook Ha[1*] and Soondo Hong[2]*

[1]Samsung Advanced Institute of Technology, Samsung Electronics Corporation, Suwon 443-803, Korea

[2]Department of Industrial Engineering, Pusan National University, Busan, 609-735, South Korea

*misook.ha@samsung.com

*soondo.hong@pusan.ac.kr

Supplementary Figure 1.

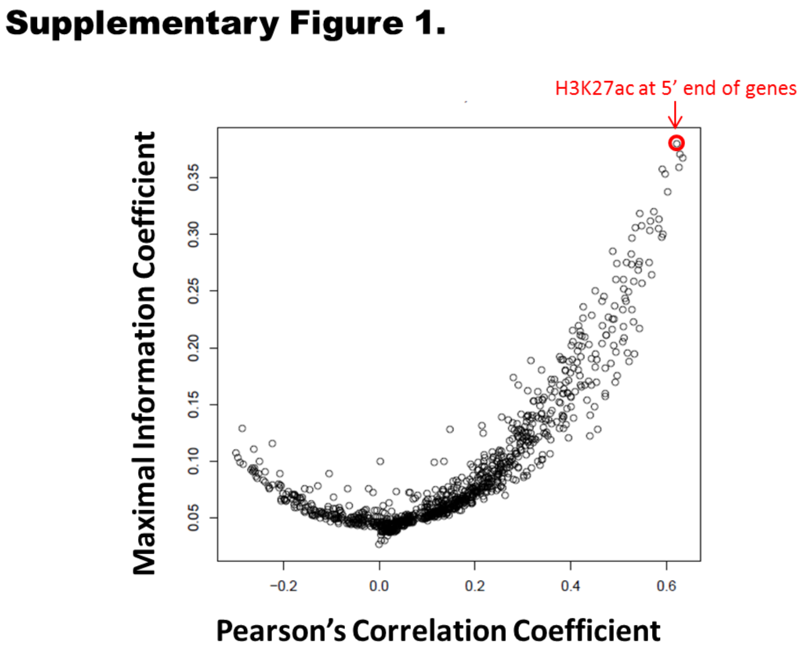

Supplementary Figure 1. Relationship between DNA binding factors and gene regulation in mESC

MIC values between DNA binding factors and transcription regulation in mESC. Linear and non-linear association measurements of position-specific protein binding and mRNA level show that H3K27ac are highly representative DNA binding features of gene regulation in coding region. Y-axis is the maximal information criterion (MIC) between protein binding intensity and mRNA levels in mESC implying linear and non-linear association. X-axis is the Pearson's correlation coefficient between protein binding and mRNA level in mESC implying linear association.

Supplementary Table 1.

DNA binding factor	Position from gene start site	MIC
H3K27ac	+200bp	0.38
E2F1	TSS	0.37
E2F1	+200bp	0.37
H3K27ac	+400bp	0.36
H3K27ac	TSS	0.36
E2f1	-200bp	0.35
E2f1	+400bp	0.34
H3K9ac	+400bp	0.32
LSD1	TSS	0.32
H3K9ac	+600bp	0.31
PolII	TSS	0.31
H3K9ac	+200bp	0.31
LSD1	-200bp	0.31
H3K9ac	+800bp	0.30
PolII	+200bp	0.30
E2F1	+600bp	0.30

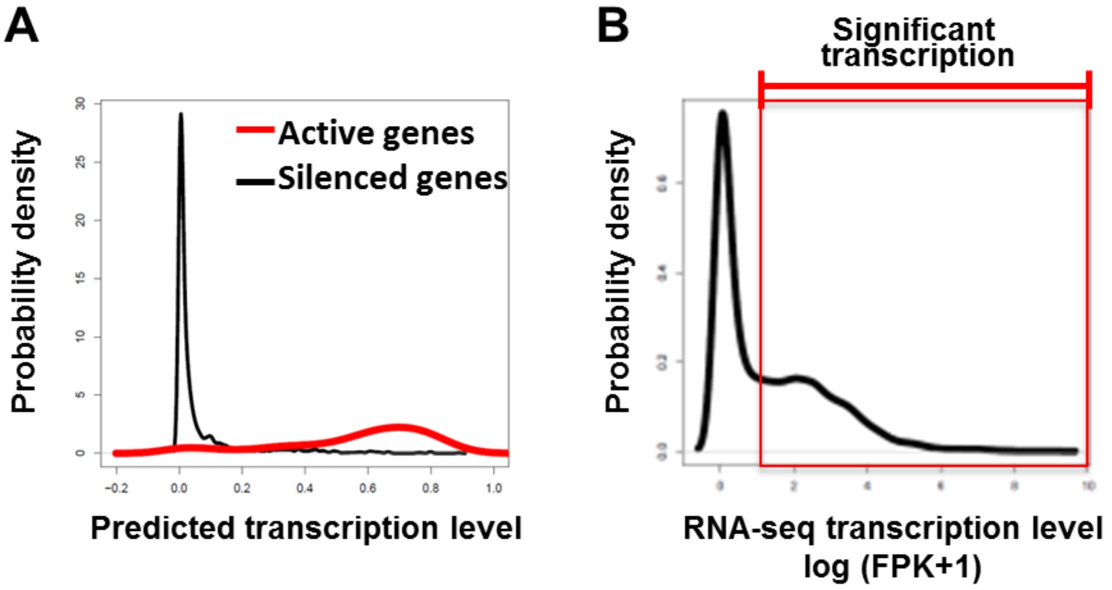

Supplementary Figure 2. H3K27ac profile predicts gene regulation in mESC.

(A) The distributions of the predicted mRNA levels in the genes significantly transcribed and silenced are consistent with the RNA-seq experiments. (B) The distribution of mRNA levels in the RNA-seq experiments in mESC. The red box shows a range of significantly transcribed gene expression levels. The horizontal axis is the log values of one plus mRNA levels measured with RNA-seq, FPKM (Fragments Per Kilobase Of Exon Per Million Fragments Mapped).

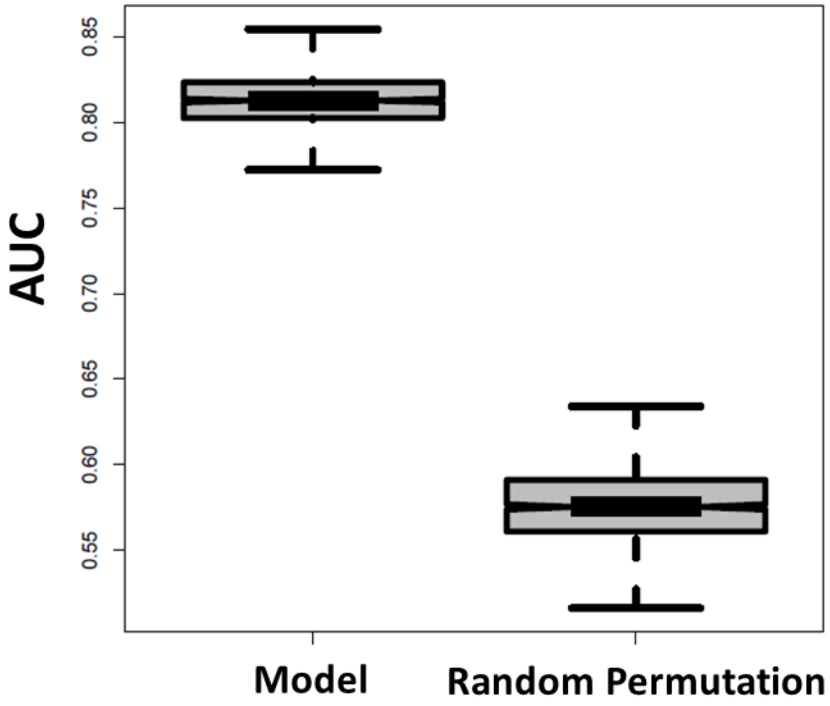

Supplementary Figure 3. Validation of predictive models of mRNA levels in mESC from H3K27ac ChIP-seq reads in TSSs.

In an ROC (Receiver operating characteristic) curve, the true positive rate (sensitivity) is plotted in function of the false positive rate (1- specificity) for different positivity thresholds of a H3K27ac enrichment. The AUC (Area Under Curve) is a measure of how well a predictive model can distinguish between genes showing significantly expressed RNA levels and not detectable RNA levels in mESC. To validate the model, we conducted the following procedures 1,000 times. (1) We randomly selected 10,000 genes as a training data set. (2) We generated a predictive model of mRNA levels from H3K27ac ChIP-seq signals of the training data. (3) We applied the model to randomly selected 1,000 genes from the remaining gene sets. (4) As a performance measure of the model, we measured AUC values of the test data set. As a negative control, AUC values are measured using random permutation of predicted values.

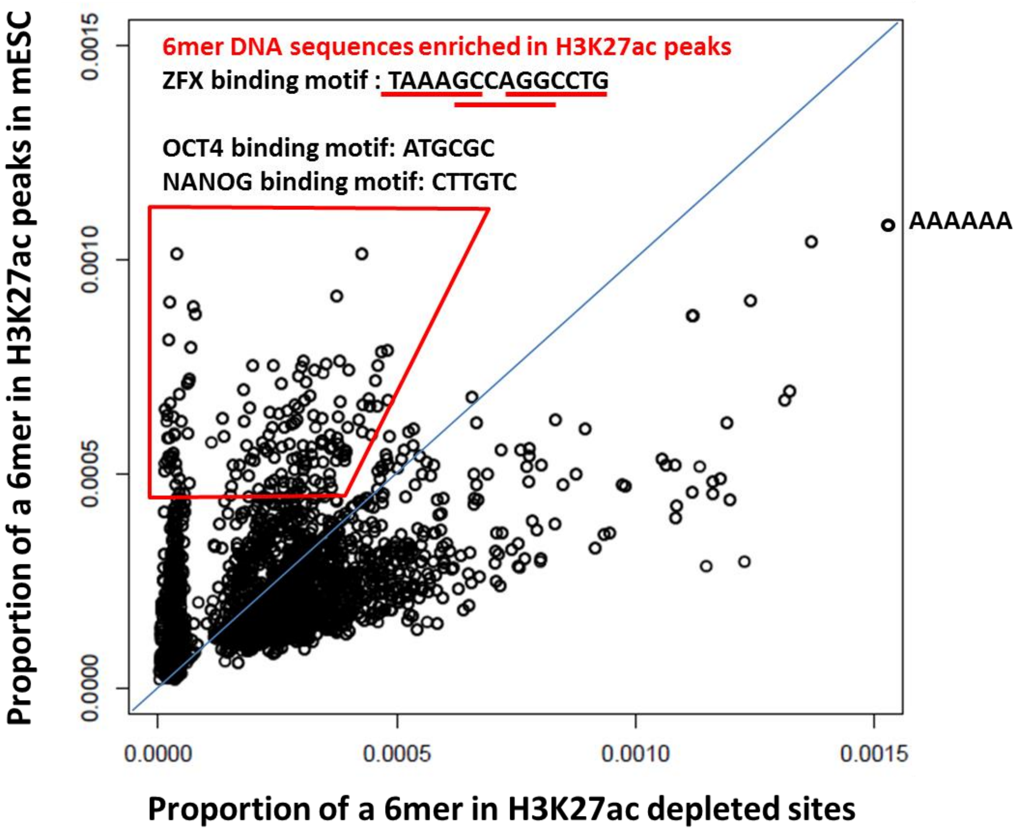

Supplementary Figure 4. Comparison of DNA 6mer sequence composition in H3K27ac peaks and H3K27ac depleted sites around TSS.

We mapped the H3K27ac specific sequences to the known TF binding motifs in the JASPAR database, which shows that the 6mer sequences specifically enriched in H3K27ac peaks around TSS match the known protein-binding motifs such as ZFX, MYC, KLF4 and OCT4. 6mer sequences significantly enriched in H3K27ac peaks around TSS include known transcription factor motifs as shown in Supplementary Table 2. The sequences associated with nucleosome-free.regions such as AAAAAA are not significantly associated with H3K27ac peaks around TSS. Horizontal axis stands for probability of observation of a 6mer in H3K27ac depleted sites around TSS and vertical axis strands for probability of observations of a 6mer in H3K27ac peaks around TSS. Each point represents a 6bp DNA sequence. The plot contains 4^6 (= 4096) points, corresponding to proportion of individual 6mer in H3K27ac peaks and depleted sites around TSS respectively. Red box indicates H3K27ac specific 6mer sequences.

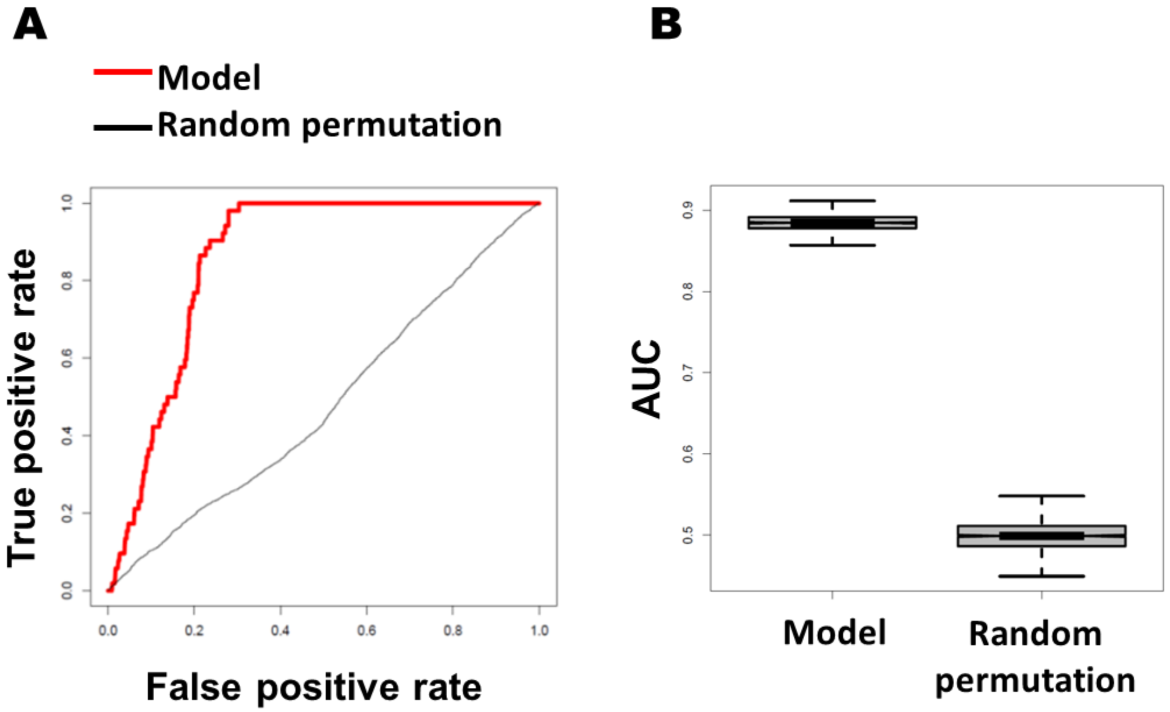

Supplementary Figure 5. Performance of H3K27ac level prediction from DNA sequences around TSS.

A. In an ROC (Receiver operating characteristic) curve, the true positive rate (sensitivity) is plotted in function of the false positive rate (1- specificity) for different positivity thresholds of a H3K27ac enrichment. The AUC (Area Under Curve) is a measure of how well a predictive model can distinguish between loci with and without H3K27ac enrichment signals. The AUC value is 0.89 with p-value < 10^{-16}. **B.** To further validate the model, we conducted the following procedures 1,000 times. (1) We randomly selected 10,000 DNA sequences as a training data set. (2) We generated a predictive model of H3K27ac from the DNA sequences of the training data. (3) We applied the model to randomly selected 1,000 DNA sequences from the remaining gene sets. (4) As a performance measure of the model, we measured AUC values of the test data set. As a negative control, AUC values are measured using random permutation of predicted values.

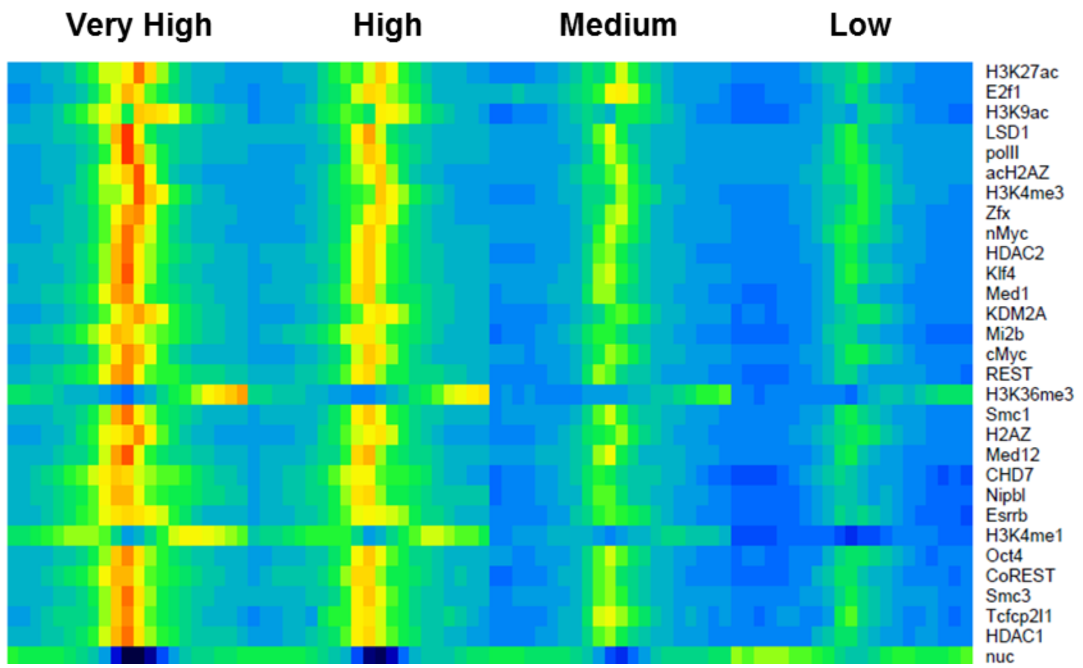

Supplementary Figure 6. High level of H3K27ac signatures inferred from DNA is associated with enrichment of transcription activator binding and depletion of nucleosomes.

Genes are classified by levels of H3K27ac signatures in TSS DNA sequences; Very high H3K27ac signatures are greater than 0.9 probability of H3K27ac peaks, high signatures are between 0.5 and 0.9, medium signatures are between 0.1 and 0.5, and low signatures are less than 0.1. The warm colors represent enrichment of ChIP-seq reads mapped, while blue color means depletion of ChIP-seq reads mapped.

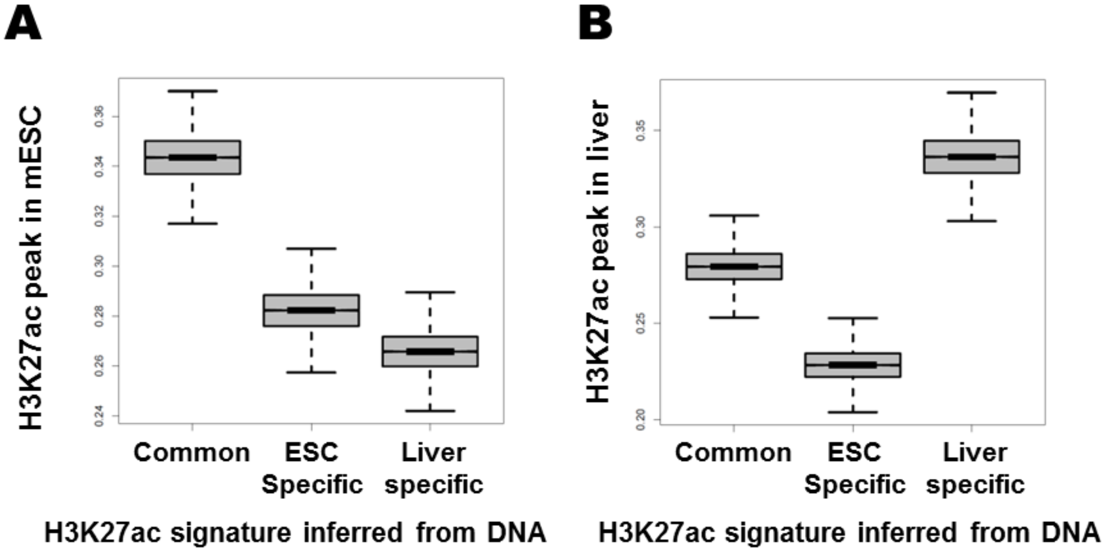

Supplementary Figure 7. The models based on differential sequence specificity of H3K27ac in mESC and mouse liver cells are correlated with cell-type specific H3K27ac peaks.

(A, B) mESC-specific signatures are preferentially found in mESC-specific H3K27ac peaks, whereas adult liver-specific signatures of H3K27ac are preferentially found in liver-specific H3K27ac peaks. The vertical axes are the H3K27ac peak heights in mESC (A) and the adult liver cells (B). The box plots show the distributions of H3K27ac peak heights in mESC (A) and adult liver (B) in common, mESC-specific and mliver-specific H3K27ac signatures in DNA.

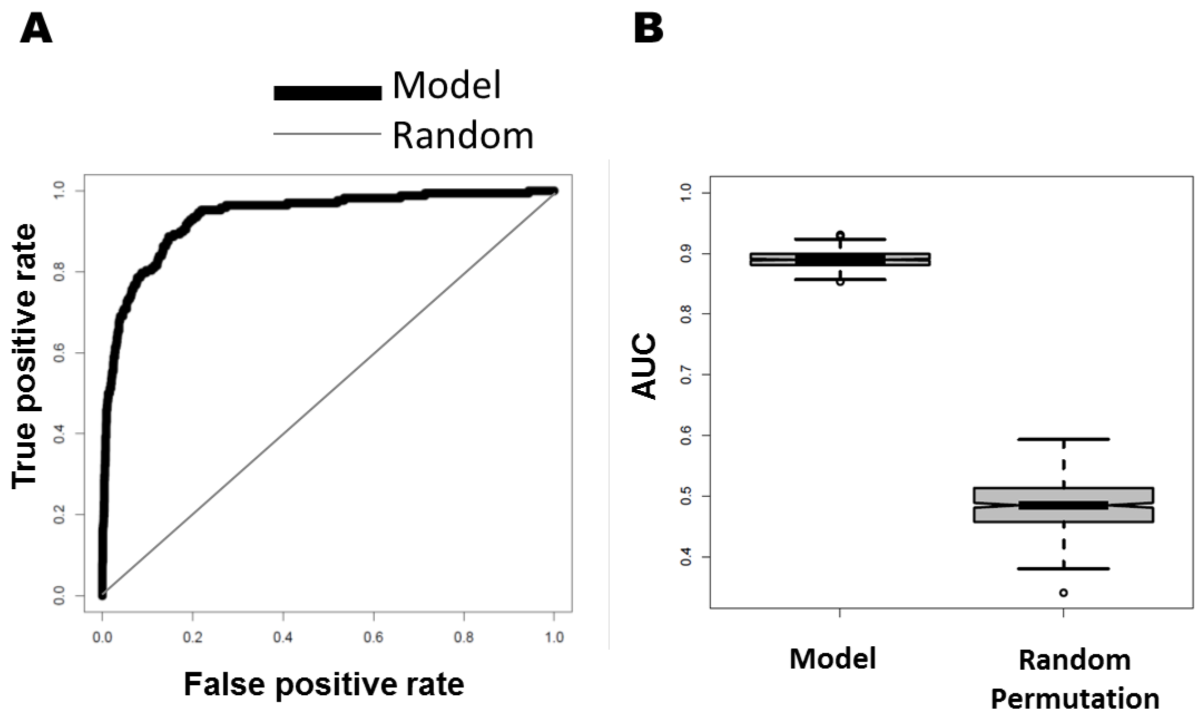

Supplementary Figure 8. Performance of gene expression inference from DNA sequence around TSS.

A. The performance of predictive model of gene expression from DNA is evaluated from the area under the ROC curve computed by genes which measure the top 20% RNA levels as positives and the genes with no detected RNAs in RNA-seq as negatives. The AUC (Area Under Curve) is a measure of how well a predictive model can distinguish between genes with and without significant expression. The AUC value is 0.93 with p-value < 10^{-16}. **B.** To validate the model, we conducted the following procedures 1,000 times. (1) We randomly selected 20,000 genes as a training data set. (2) We generated a predictive model of H3K27ac from the DNA sequences of the training data. (3) Using the predicted profile of H3K27ac from the DNA sequences, we generated a model predicting RNA levels in the training gene set. (4) We applied the model to randomly selected 100 genes from the remaining gene sets. (5) As a performance measure of the model, we calculated AUC values by comparing the predicted and measured RNA levels of the test data set. We performed 1,000 repetitions of the above procedures and obtained 1,000 measurements of the AUC values as model performance. As a negative control, AUC values are measured using random permutation of predicted values.

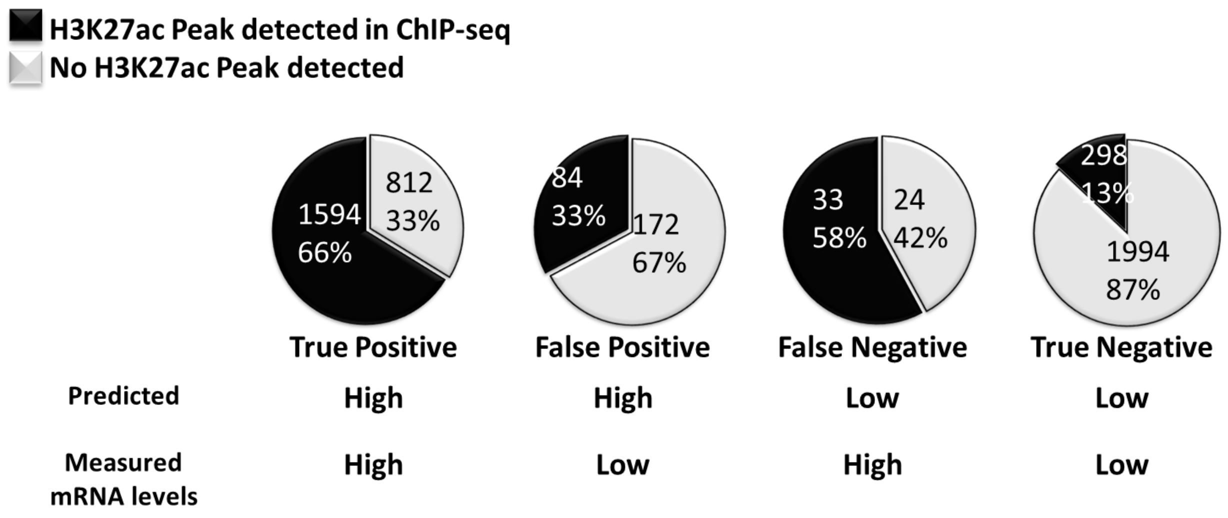

Supplementary Figure 9. Association of the inferred gene expression with H3K27ac ChIP-seq peaks around TSS.

The pie charts show numbers and proportion of genes associated with H3K27ac ChIP-seq read enrichment (black) and no H3K27ac enrichment (gray) in 2kbp regions around TSS. Top 10% (2660) highly expressed genes in mESC are regarded as positive and gene transcripts not detected in RNA-seq are regarded as negative. Genes are classified into 4 categories: (1) True positive prediction of gene expression; (2) false positive prediction of gene expression; (3) false negative prediction of gene expression; and (4) true negative prediction of gene expression. Genes in false positive prediction of expression are significantly more enriched with genes not associated with H3K27ac ChIP-seq enrichment around the TSS (Chi-squared test, Chi-squared = 109.6, p-value < 10^{-16})
